# Workability in the Ageing Workforce—A Population-Based Cross-Sectional Study

**DOI:** 10.3390/ijerph182312656

**Published:** 2021-11-30

**Authors:** Niels-Peter Brøchner Nygaard, Gert Frank Thomsen, Jesper Rasmussen, Lars Rauff Skadhauge, Bibi Gram

**Affiliations:** 1 Research Unit of Health Science, Hospital of South West Jutland, University Hospital of Southern Denmark, 6700 Esbjerg, Denmark; Bibi.valgerdur.gram@rsyd.dk; 2 Department of Regional Health Research, University of Southern Denmark, 5230 Odense, Denmark; lars.rauff.skadhauge@rsyd.dk; 3 Department of Occupational Medicine, Hospital of South West Jutland, University Hospital of Southern Denmark, 6700 Esbjerg, Denmark; gert.frank.thomsen@rsyd.dk; 4 Department of Occupational and Environmental Medicine, Odense University Hospital, 5000 Odense, Denmark; Jesper.rasmussen5@rsyd.dk; 5 Department of Clinical Research, University of Southern Denmark, 5230 Odense, Denmark

**Keywords:** work demands, ergonomic exposure, aging, musculoskeletal pain

## Abstract

Background: The purpose of this study was to investigate the impact of age, musculoskeletal pain and ergonomic exposure on workability in the oldest group of workers. Methods: The study was a population based cross-sectional survey. The study population comprised citizens born between 1952–1966, living in Esbjerg municipality ultimo 2016 (*n* = 23,463). A questionnaire was sent electronically or by mail. The analysis included the working population only. A stereotype logistic regression was used with the primary dependent variable being workability and independent variables included age, musculoskeletal pain, and ergonomic exposure. Results: The response rate was 58% and the data demonstrated a significant negative association between age and workability. With excellent workability as a reference, the odds for poor workability increased by 97% being 60+ y compared to 50–55 y. Both moderate intensity and severe musculoskeletal pain in the back, shoulder and knee/hip all showed significantly higher odds for poor workability. Ergonomic exposures, such as standing/walking, working with back bent or twisted and carrying or lifting had a significant negative impact on workability. Conclusion: Age, musculoskeletal pain and ergonomic exposures showed a significant negative impact on workability in the oldest group of workers and should be targeted with preventive initiatives.

## 1. Introduction

The size and composition of the workforce has changed dramatically in recent decades [1]. The increased proportion of the population above 55 years combined with increased retirement age to meet socioeconomic demands, might have unforeseen consequences for the workability in this age group [2]. Aging is associated with a decline in both physical and mental health [3] and studies show a significant impact on muscle strength [4,5], bone density and aerobic capacity, which are dependent on lifestyle, body weight and genetics [6]. As a consequence, older persons might not be able to adequately fulfill the work-related physical demands that are required and research suggests increased risks for disability [7], occupational injury [8], musculoskeletal disorder [9] and early retirement [10]. There is, therefore, a need for increased focus on individuals’ capacity to cope with various job exposures as individuals age and on its consequences for individuals’ workability and health. This would allow clinicians within occupational medicine to better diagnose and treat patients in an already challenging domain. 

The term workability has proved useful to address several work and lifestyle-related factors [11] and is commonly evaluated using the Work Ability Index (WAI) [12,13]. Workability is multifactorial and reflects the balance between work demands and the worker’s capacity to cope with those demands. A number of factors have been shown to affect workability, such as age [14], health problems [15], physical capacity [11], obesity [16], work type [7] and leisure physical activity [17]. This suggests a complex interaction between human resources and work that continues to change as people age. Musculoskeletal disorders, (MSD) has been shown to be more prevalent in the older workforce compared to younger workers and have been associated with an increased risk of sickness absence [18,19], musculoskeletal pain [20] and other comorbidities [21,22]. Across the member states of the EU, the rate of MSD in 2013 was 60.1% and in the US labor force MSD was the most commonly reported type of illness in 2010 [23]. Musculoskeletal pain and comorbidities might further negatively interact, exacerbating the impact on work ability, quality of life and mortality [24,25]. 

Previous studies have focused on the differences in the older workforce above 50 y and compared younger age groups [14,22,26,27] showing that age might be one of the more important predictors for poor workability alongside pain. Others show that age might not be as important [27] or that the imbalances caused by a loss in physical capacity or musculoskeletal disease may be partially compensated for by other resources [28]. Importantly, age has been shown to be more critical in jobs with high physical demands [29] since it is not possible in most cases to compensate for the loss in physical function to fulfill requirements in these jobs. Continuous exposure to ergonomic loads is likely to increase the risk for musculoskeletal disorders and poor workability outcomes [30]. Consequently, studies have shown the excess risk for musculoskeletal disorders in health care workers [31], manufacturing and industrial work [32,33], and in construction [34] to name a few. Nevertheless, while research has been done on this topic, it fails to capture any changes in workability in individuals specifically during the last years on the labor market—losing valuable information that might help guide future health promotion and prevention. The time period above 50 years, is characterized by significant physical changes, particularly after 60 years showing a more rapid decrease in functional capacity [35] and a higher prevalence of health issues [36], which may have a significant impact on workability. The large majority of studies focusing on workplace interventions show only small positive effects [37] indicating a need for additional high quality studies elucidating the dynamic and intensive interaction between human resources and work, which is particularly complex in the oldest workers. Increased knowledge on how the possible association and interaction between work exposure and physical health change in the oldest working population is key to ensure worker wellbeing and workability, as well as their continued attachment to the labor market. This would also enable targeted preventive initiatives and treatment among older workers within the frame of occupational medicine. 

To gain knowledge on the health and workability-related consequences of a continuingly prolonged work life, we established a representative cohort of an aging population (≥50 y). The purpose of the present cross-sectional study was to investigate the impact of age, musculoskeletal pain and ergonomic exposure on workability in the oldest group of workers. We hypothesized that age, independently of other variables, would be negatively associated with workability. This would also be the case for musculoskeletal pain and ergonomic exposure. 

## 2. Materials and Methods

### 2.1. Study Design

The present study is a population-based cross-sectional survey conducted in the fourth quarter of 2017—second quarter of 2018 in the Esbjerg municipality. A comprehensive questionnaire was constructed based on items previously used in occupational health research, with emphasis on health status, musculoskeletal disorders, perceived stress, ergonomic exposure and workability. This study presents the first results in an established cohort that is to be followed prospectively. Follow-up is planned for 2023–2024. The present study focused only on the impact of age, musculoskeletal pain and ergonomic exposure on workability. The study follows the guidelines for the reporting of observational studies in epidemiology (STROBE).

### 2.2. Ethics

The study was registered with The Danish Data Protection Agency (file no. 2008-58-0035). In accordance with Danish law and the Regional Committees on Health Research Ethics for Southern Denmark (file no. S-20180162), questionnaires and register-based studies of the type presented here does not need approval. Data were anonymized and analyzed based on code identifiers. 

### 2.3. Participants

The study population comprised citizens born between 1952 and 1966 living in the Esbjerg municipality in December 2016 (*n* = 23,463). Names and social security numbers of the population were obtained from the Danish Health Data Authority. When a study is accepted by the Danish Data Protection Agency, the Danish Health Data Authority can deliver data for research purposes after approval of the protocol. A questionnaire was sent electronically to their public electronic mailbox (eBoks); eBoks is a secure electronic online mailbox used to receive digital mail from the private sector and from the public authorities by syncing your eBoks with your Digital Post account. Citizens without eBoks received the questionnaire by mail. The Danish Health Data Authority supplied the mail addresses for these citizens. In case of non-response, the questionnaire was sent once again, regardless of the delivery form. A total of 58% responded to the questionnaire. Data were collected using the REDCap electronic data capture tool (OPEN, University of Southern Denmark) [38]. The present study included individuals that reported to be employed or self-employed when answering the questionnaire.

### 2.4. Outcome Variable

The outcome variable was workability measured using a single-item instrument. Respondents were asked to rate their workability on a visual analog scale from 0–100, where 0 was “Not able to work” and 100 was “Best imaginable workability”. The scores were then categorized into poor (0–50), moderate (51–70), good (71–90) and excellent (91–100) workability, as suggested by the original authors [39].

### 2.5. Predictor Variables

#### 2.5.1. Age

Respondents were classified into three different age groups: 50–55, 56–60, and >60 years.

#### 2.5.2. Musculoskeletal Pain

The Standardized Nordic Questionnaire (SNQ) [40] was used to assess musculoskeletal pain and has been used extensively within the context of occupational health. The present study focused on pain in the body regions: back, shoulder and hip/knee. We used an average pain score for the past 3 months, as measured by the visual analog scale (VAS), where 0 was defined and labeled as “no discomfort” and 100 as the worst imaginable pain and discomfort for each region. The scores were categorized into low (VAS 0–30), moderate (VAS 31–60) and severe pain (VAS 61–100). 

#### 2.5.3. Ergonomic Exposure

Estimation of physical work demands was assessed with eight questions: During the working day—to which extent do you: (a) sit, (b) walk or stand, (c) work with your back strongly bent forward without hand- and arm support, (d) have your arms raised to or above shoulder height, (e) perform repetitive arm movements several times per minute (e.g., package work, mounting, machine feeding, carving), (f) squat or kneel when you work, (g) push or pull, (h) carry or lift. The answer categories were: (1) almost all the time, (2) approximately ¾ of the time, (3) approximately ½ of the time, (4) approximately ¼ of the time, (5) rarely/very little, or (6) never. The questions were further categorized into low (5, 6), moderate (3, 4) and high exposure (1, 2) respectively. Question a was left out of the analysis since it was an antagonist to question b. 

### 2.6. Control Variables

A range of potential covariates were identified and controlled for in the analysis. Gender and body mass index (BMI) has been shown to affect workability [16]. BMI was calculated using the respondents’ weight in kilograms divided by the square of height in meters (kg/m^2^), and categorized into underweight (<18.5), normal (18.5–24.9), overweight (25.0–29.9), obese (30.0–34.9) and extremely obese (>40.0). Smoking status also might affect workability [14]. Smoking was assessed with the question: “Do you smoke tobacco” with the following categorical variables “Yes”, “No”, and “Previously”. Work-related stress was assessed using the Danish version of the 10-item Perceived Stress Scale (PSS) using a sum score. Leisure time physical activity at moderate intensity also have a significant impact on workability as demonstrated by Norheim et al. (2020) [41]. Participants were asked to describe their leisure physical activity for: (a) recreational sports, heavy gardening, or fast walking/cycling where you sweat or get short of breath, b) high intensity training or competitive sports, in terms of (1) does not perform the activity, (2) under 2 h per week, (3) 2–4 h per week and (4) more than 4 h per week. The scores were dichotomized into no physical activity (1–2) or physically active (3–4). Finally, chronic disease including cardiovascular disease, cancer, diabetes, depression, asthma, chronic obstructive pulmonary disease, metabolic disease was assessed with the categorical options “Yes” and “No” [15].

### 2.7. Statistical Analysis

The analyses and statistics were performed using the statistical software STATA16 (StataCorp LLC, College Station, TX, USA). Descriptive statistics of the population are presented as frequencies (*n*) and percent of the total population (%) included in the study. Stereotypic logistic regression was used to estimate the associations between workability (dependent variable) and age, ergonomic exposure, musculoskeletal pain (independent variables). The model was adjusted for sex, BMI, leisure time physical activity, smoking, work-related stress, and chronic disease. Results are reported as Odds Ratio (OR) and 95% confidence intervals (CI) unless otherwise stated. The model did not impute missing values. The effect size was estimated using Chen et al. 2010 [42]; small effect size: OR < 1.6, medium effect size: OR > 1.5|OR < 5, large effect size: OR > 5. 

## 3. Results

**Figure 1:**
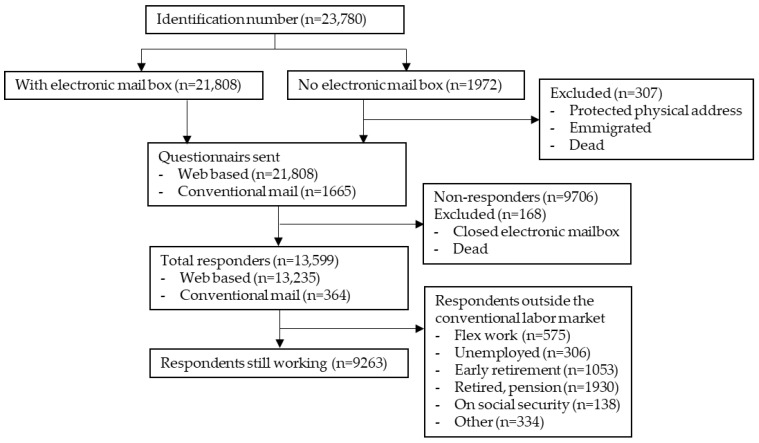
Flow diagram. Depicts the number of individuals identified in the Esbjerg municipality, the number of respondents to the questionnaire and the number of respondents included in the study.

In total, 23,780 citizens at age 50–64 were identified in the Municipality of Esbjerg in December 2016 and among these, 21,808 had a valid eBoks and received a web-based questionnaire (Figure 1). From the remaining 1972 persons, it was possible to retrieve a valid postal address for 1655 persons from Statistics Denmark. Eleven persons had emigrated, two had disappeared, one person changed identity, ten were unknown at the address, 13 had protected address and 280 had passed away before retrieval of the postal addresses leaving a total of 23,463 persons eligible for the study (Figure 1). After one reminder 13,599 (58%) had answered the questionnaire. Of these, 9263 (68%) stated to be at work when answering the questionnaire. In Esbjerg Municipality 65% of the population aged 50–64 were at work [43], showing a very modest over-representation in the responders. The demographics of the population are presented in Table 1. 

The results from the stereotype logistic regression are presented in Table 2.

### 3.1. Age

With excellent workability as the reference and adjusted for all other variables, the odds for poor workability were significantly higher comparing the older age groups 56–60 and 60+ with the younger 50–55 age group. For the group aged 56–60, the odds for poor workability were 1.44 times higher than the 50–55 age group. The OR for poor workability was highest in the oldest group of workers, showing 1.97 higher odds than the 50–55 age group (Table 2). 

Evaluating the OR for the other categories of workability, i.e., moderate and good versus excellent, the 56–60 age group and the 60+ age group showed significantly higher odds for lower workability compared to the younger 50–55 age group. The odds for moderate workability were 1.34 times higher in the group aged 56–60 y compared to the 50–55 y age group and 1.72 times higher in the age group 60+. Finally, the odds for good workability were 1.17 times and 1.35 times higher in the 56–60 age group and 60+ age group respectively, compared to the 50–55 age group, adjusted for all other variables (Table 2).

### 3.2. Musculoskeletal Pain

With excellent workability as the reference and adjusted for all other variables, moderate-intensity and severe musculoskeletal pain in the back, shoulder or hip/knee resulted in significantly higher odds for poor workability compared to having low-intensity pain (Table 2). Higher reported pain intensities, in either region, resulted in significantly higher odds for poor workability.

Moderate intensity pain in the back compared to low-intensity pain, resulted in 2.08 times higher odds for poor workability, adjusted for all other variables. Having severe pain in the back, again compared to low-intensity pain, resulted in 2.85 times higher odds for poor workability. Similar results were observed evaluating the ORs for moderate and good workability, albeit overall lower (Table 2).

Similar to back pain, moderate-intensity pain in the shoulder compared to low-intensity pain, resulted in 1.83 times higher odds for poor workability, adjusted for all other variables. Severe pain in the shoulder, also compared to low-intensity pain, resulted in 3.29 times higher odds for poor workability. Looking at the ORs for moderate and good workability, revealed overall lower but similar results. 

Finally, adjusted for all other variables, moderate-intensity pain in the hip/knee was compared to low-intensity pain and resulted in 1.61 times higher odds for poor workability. Comparing severe pain in the hip/knee with low-intensity pain, revealed 3.47 times higher odds for poor workability and similar results were observed when evaluating the ORs for moderate and good workability, still with excellent as the reference. 

### 3.3. Ergonomic Exposure

A variety of ergonomic exposures were investigated in the present study and three showed statistically significant changes. Excellent workability was used as the reference. Working postures including walk/stand showed varying results across the workability score. High exposure of work-related walk/stand compared to low exposure showed 1.73 times higher odds for poor workability while showing 0.8 times higher odds for moderate workability and 1.27 times higher odds for good workability, adjusted for all other variables. 

Being exposed to work with the back twisted/bent showed that even moderate exposure, compared to low, resulted in 1.50 times higher odds for poor workability. Looking at high exposure of working with to the back twisted/bent, again compared to low, resulted in 1.88 times higher odds for poor workability, adjusted for all other variables. 

Similarly, having excellent workability as the reference and adjusted for all other variables, the odds for moderate and good workability were significantly higher for those who were moderately and highly exposed to risk factors, such as back twisted/bend compared to low exposure of these factors (Table 2). 

Finally, being moderately exposed to working postures involving carrying or lifting, resulted in 2.01 times higher odds for poor workability compared to low exposure. Still, when comparing moderate to low exposure to carrying or lifting the results revealed significantly higher odds for both moderate and good workability. 

Working with arms above shoulder height, repeated arm movement, squatting or lying on knees, or pushing or pulling, did not show any statistically significant results.

## 4. Discussion

The aim of the present study was to investigate the impact of age, musculoskeletal pain and ergonomic exposure on workability in the oldest group of workers. Adjusted for all other variables, age was negatively associated with workability showing higher odds for poor workability compared to excellent in the older age groups. Pain in the back, shoulder, or knee/hip also showed significantly higher odds for poor workability, as did exposure to standing, back twisted/bend and carrying or lifting. The stereotype logistic regression model used in the present study allowed for a comparison between different workability groups and demonstrated that important changes occurred across all groups, with a significant decrease from excellent workability to a concomitant increase in good, moderate and poor workability. Importantly these changes occurred across a relatively narrow age span underlining their clinical significance. 

### 4.1. Age

The present study showed that workability was significantly affected in the oldest working population. Being 55+ years, independent of other variables, resulted in significantly higher odds for poor workability compared to excellent. Specifically, with excellent workability as a reference, the odds for poor workability nearly doubled being 60+ compared to 50–55 y, underlining the importance age has on workability. The results were in line with previous findings, for example, Pohjonen et al. compared workability in different age groups of home care workers and found that workability significantly decreased at 40–44 y of age and with a further steep decrease after 55 y of age. They also noted that the number of workers having poor workability increased three-fold in the oldest age group (55–62 y) compared to the younger age groups [44]. Similarly, El Fassi et al. examined workers aged 40 to 65 occupying a wide variety of job functions and showed a strong negative association between age and workability, with increasing odds for poor/moderate workability as age increased [14]. These, as in numerous other studies [22,36,45,46], support the findings in the present study demonstrating that younger workers report higher workability than older workers, in a variety of workers. In contrast to earlier studies, the present study demonstrated a significant negative impact on workability across a relatively short age span. This highlights an increased negative impact on workability during the last 10 years of work, independently of other variables, warranting increased focus on this age group both clinically and at the workplace. 

Most studies chose a cut-off on the workability score, and thus, compares good-excellent with poor-moderate, risking a significant loss of information. Even the more subtle and statistically significant change, comparing good and excellent workability, as demonstrated in the present study, might have significant prognostic and clinical implications. Studies show that having less than excellent workability was a significant predictor for sick leave in construction workers [47] and in a representative sample of the Danish working population, Sell et al. showed that a 1 point decrease in perceived workability on a 10-point scale, was associated with increased risk of early retirement (33%) and long-term sickness absence (15.1%) [48]. It could be speculated that the impact of a 1-point decrease in workability would be even higher in an older working age group and that the impact of having poor workability, would be even more detrimental. Poor workability has been shown to be a predictor for early retirement [49], sickness absence, work stress [50], depression [51] and others [22]. 

Aging has been associated with significant changes in both physical and mental capacity and if work demands exceed physical and mental capacity workers might experience health problems. The changes observed in the present study underline that age, independently of other variables, imposes important clinical implications. 

The significant association between age group and workability might indicate that the demands of the job are not in agreement with age-related changes in mental and particularly physical capacity. To investigate this further, the present study hypothesized that age would act as an effect modifier for, for example, ergonomic exposure. Interaction effects (age group*ergonomic exposure) were thus included and tested in the regression model but did not show any statistically significant results. A healthy worker effect might explain why this was not the case. Individuals, who experience difficulties in coping with high demands, may have retired early, as suggested previously [14]. 

### 4.2. Musculoskeletal Pain

Moderate intensity pain and severe pain in the back, shoulder, or hip/knee region resulted in significantly higher odds for poor workability compared to low-intensity pain. Although, some studies have reported that workability can be maintained despite musculoskeletal pain [52] the majority of studies [30,53,54], are in line with the present study showing a significant impact of pain on workability. Most of these studies show an impact on workability due to pain at multiple locations and other studies have shown that pain at a single location had a much lower negative effect [55,56]. This study demonstrated a clear association between single-site pain in the back, shoulder, or hip/knee with lower workability, independently of other variables. 

Back, shoulder and hip/knee pain are among the most common musculoskeletal complaints [57] and are often work-related [58]. Risk factors include ergonomic exposures and previous findings suggest a strong association between ergonomic exposure and the development of musculoskeletal disorders. For example, Miranda et al. reported that the risk of developing shoulder disorders increased by 80–150% when exposed to ergonomic strain [59]. Other risk factors include psychological state and demands [60], as well as psychosocial factors [61]. The present study showed that the impact of pain on workability was important at both moderate and particularly severe pain intensity levels. In this line, pain intensity levels have previously been shown to be a negative prognostic factor [62] and the individual’s ability to carry out daily activities and work is significantly affected [63]. Further studies are needed to investigate the interplay between physical and psychosocial demands and their impact on musculoskeletal pain. Finally, pain has been associated with higher risks of both sick leave and disability pension [54,64]. Chronic pain affects a large proportion of individuals and thus, a significant fraction of the working population. It is thus imperative to find and implement strategies that take musculoskeletal pain into consideration, by balancing physical and psychological strain.

### 4.3. Ergonomic Exposure

Workers with high physical work demands have been shown to be at risk of disability, and a negative association between workability and work demands has been demonstrated in the older age groups. The present study demonstrated that, independently of other variables, walking/standing for more than 75% of the workday results in significantly higher odds for poor workability compared to excellent. This result is less clear since the odds for moderate workability are lower compared to excellent and higher for good workability compared to excellent. These discrepancies were likely explained by having both standing and walking in the same category since these might in part counteract each other [65]. Future studies should try to separate these categories. Standing, in particular, has previously been associated with important health risks, such as cardiovascular problems, MSDs and long-term sick leave [64,66] and have been reported to reduce blood supply to the muscles, accelerating fatigue, discomfort and changing the activity of the muscles as well as postural stability [65,67]. On the other hand, studies have also shown detrimental health effects of prolonged sitting [68] and beneficial effects of sit-stand workstations [69]. In addition to ergonomic exposure to standing/walking, the results in the present study indicated that working with the back bent or twisted was one of the primary predictors for poor workability. Even moderate exposure (25% of the workday) to working with the back bent or twisted, resulted in significantly higher odds for poor workability, and high exposure (75% of the workday) had an even more pronounced negative impact on workability. In line with this finding, Skovlund et al. also showed a significant association between working with the back twisted or bent and poor/moderate workability in a population with pain in the hand/wrist, neck/shoulder and low-back region [30]. Sterud et al. also reported similar findings predicting long-term sick leave, also showing a significant effect of back bent or awkward lifting on workability [64]. Moderate exposure to carrying or lifting was also a significant predictor for poor workability in the present study. The present study did not find an association between repetitive arm movement and squatting or lying on knees which have been observed by others [30,64,66,70]. For example, repeated arm movement was associated with long-term sickness for blue collar workers in Andersen et al. [71] but not in the general population. Sterud et al. observed that both repetitive arm movement, standing and squatting or kneeling were associated with long-term sick leave [64]. This is in contrast to the current study and might suggest both a difference in methodology and that other factors in combination with ergonomic exposure need to be present to have a detrimental effect on workability. 

The oldest age group +60 y has shown an important decrease in muscle strength [72] and cardiorespiratory fitness [41,73], which could influence the feasibility of certain ergonomic exposures. Poor work conditions have been found to be a significant predictor for poor workability in previous studies [11,44,74] and estimates of 21–34% of disability pension cases have been shown to be attributable to ergonomic exposures at the workplace [75]. The ergonomic risk factors demonstrated in the present study, i.e., standing/walking, back twisted/bent and carrying or lifting, should be the primary focal point for future intervention studies, although further studies are needed to further specify potential risk factors. These exposures were also demonstrated to be highly prevalent [76] and associated with serious disease [77] and musculoskeletal pain [78] highlighting their clinical importance. There is a clear association between ergonomic exposure and musculoskeletal pain and musculoskeletal pain is often the primary reason for seeking medical advice.

### 4.4. Strengths

The cross-sectional design in the present study allows for multiple outcomes to be studied, which might be used for generating further hypotheses and in-depth research studies. It also allows for the adjustment of several covariates, that might act as confounders or effect modifiers.

The response rate in the present study was good (58%), relative to similar studies, and the sample was large and representative of the working population. This strengthens the statistical power of the current study considerably. The study uses the workability score, which is a validated instrument, that is both useful and practical in a clinical setting. Finally, using stereotype logistic regression, it was possible to differentiate effects on workability across multiple categories, i.e., excellent, good, moderate and poor workability.

### 4.5. Limitations

However, the study also has some limitations. The responses in the present study might exclude the most vulnerable individuals already outside of the labor market, causing a “healthy worker effect”.

Workability is commonly assessed using the workability index. This study used the corresponding instrument with a single-item question—the workability score. Good reliability has been shown between the two instruments although differences might occur. For example, Roelen et al. compared both the index and the single-item instrument and were only able to identify workers at risk of disability pension using the index [79]. Nevertheless, the single-item instrument has been shown to be a practical and valid indicator of workability in previous studies [14,80]. 

The ergonomic exposures included in the present study were self-reported and estimates may, therefore, be influenced by symptoms or knowledge of disease status causing inflated estimates [81].

### 4.6. Practical Implications 

Recognizing the factors that influence workability is crucial to help prioritize preventive initiatives for workers at risk and for the retention of workers. Workplace interventions are complex and need to take into account the multifactorial relationship between workers and their physical and psychosocial environment. Interventions are thus, challenging to implement, and properly targeted interventions are a prerequisite. The significant predictors identified in the present study are key for such targeted interventions, which should focus on the oldest group of workers in particular.

Better knowledge of the predictors for poor workability might also allow for better clinical classification of patients in occupational medicine. This includes an improved definition of the criteria required to identify a disorder and its attribution to the workplace. This allows for better communication of prevalence and incidence rates, as well as of the impact of various disorders and their prognosis.

The results might also provide better insight into disease etiology as well as into the current experience of the patients and of the patients’ course of treatment. By evaluating the results in the present study and the literature it is apparent that clinicians need to have a multimodal approach to treatment, focusing on early return to work/retention taking both the work-related consequences of aging into account and the appertaining biological and psychosocial obstacles. In this respect, the current study showed that the oldest age group was not able to compensate for the changes occurring during aging or when in pain. Previous studies have suggested that the changes in physical capacity can in part be compensated for by increased experience, competencies, and a change in perspective. If that is to be the case, targeted interventional strategies are needed. 

## 5. Conclusions

The present study showed that workability is negatively affected in the oldest group of workers. Musculoskeletal pain and ergonomic exposures, such as standing, working with a bent back and lifting, are important negative predictors for workability. Independently of these variables, age has a large negative impact on workability during the last 10 years in the workforce. Initiatives are needed to accommodate these changes in the future. 

## Figures and Tables

**Table 1:** Descriptive statistics of the study population—citizens above 50y living in the Esbjerg minucipality in December 2016.

Variable	Frequency (n)	Percentage (%)
Gender		
Male	4681	50.5
Female	4582	49.5
Age group		
50–55 y	3253	35.1
56–60 y	3931	42.4
>60 y	2079	22.4
Workability		
Poor	384	4.1
Moderate	522	5.6
Good	3104	33.5
Excellent	3562	38.5
Back pain		
Low instensity	5384	58.1
Moderate intensity	1943	21.0
Severe intensity	686	7.4
Shoulder pain		
Low instensity	5922	63.9
Moderate intensity	1705	18.4
Severe intensity	507	5.5
Hip/knee pain		
Low instensity	6540	70.6
Moderate intensity	1493	16.1
Severe intensity	574	6.2
BMI		
Underweight	48	0.5
Normal	3095	33.4
Overweight	3484	37.6
Obese	1673	18.1
Extremely obese	142	1.5
Smoking		
Yes	1457	15.7
Previously	1747	18.9
Never	5330	57.5
Chronic cardiovascular disease		
Yes	350	3.8
Diabetes		
Yes	452	4.9
Asthma		
Yes	776	8.4
Metabolic disease		
Yes	500	5.4
Depression		
Yes	216	2.3
Cancer		
Yes	701	7.6
COPD		
Yes	285	3.1

**Table 2:** Odds ratios (OR) and 95% confidence intervals (95% CI) for poor, moderate and good work ability score (WAS) compared to excellent of employed individuals for agegroup, musculoskeletal pain and ergonomic exposure.

Variable	Good		Moderate		Poor	
	OR (95% CI)		OR (95% CI)		OR (95% CI)	
Age group						
50–55 y (n = 3253)	1	(1–1)	1	(1–1)	1	(1–1)
56–60 y (n = 3931)	**1.17**	**(1.06–1.30)**	**1.34**	**(1.11–1.61)**	**1.44**	**(1.15–1.81)**
>60 y (n = 2079)	**1.35**	**(1.19–1.52)**	**1.72**	**(1.38–2.14)**	**1.97**	**(1.50–2.59)**
Back pain						
Low intensity (n = 5354)	1	(1–1)	1	(1–1)	1	(1–1)
Moderate intensity (n = 1943)	**1.38**	**(1.24–1.54)**	**1.80**	**(1.47–2.20)**	**2.08**	**(1.62–2.68)**
Severe intensity (n = 686)	**1.58**	**(1.33–1.88)**	**2.31**	**(1.69–3.16)**	**2.85**	**(1.93–4.20)**
Shoulder pain						
Low intensity (n = 5922)	1	(1–1)	1	(1–1)	1	(1–1)
Moderate intensity (n = 1705)	**1.31**	**(1.16–1.47)**	**1.62**	**(1.31–2.02)**	**1.83**	**(1.40–2.39)**
Severe intensity (n = 507)	**1.69**	**(1.40–2.03)**	**2.60**	**(1.86–3.63)**	**3.29**	**(2.18–4.98)**
Hip/knee pain						
Low intensity (n = 6540)	1	(1–1)	1	(1–1)	1	(1–1)
Moderate intensity (n = 1493)	**1.23**	**(1.10–1.39)**	**1.47**	**(1.19–1.81)**	**1.61**	**(1.24–2.10)**
Severe intensity (n = 574)	**1.73**	**(1.43–2.09)**	**2.71**	**(1.92–3.83)**	**3.47**	**(2.26–5.33)**
Walk/stand						
Low exposure (n = 999)	1	(1–1)	1	(1–1)	1	(1–1)
Moderate exposure (n = 4038)	1.13	(0.97–1.31)	0.65	(0.48–0.87)	1.32	(0.93–1.86)
High exposure (n = 3667)	**1.27**	**(1.08–1.50)**	**0.80**	**(0.66–0.98)**	**1.73**	**(1.18–2.53)**
Back twisted/bend						
Low exposure (n = 5504)	1	(1–1)	1	(1–1)	1	(1–1)
Moderate exposure (n = 2259)	**1.20**	**(1.06–1.35)**	**1.39**	**(1.11–1.73)**	**1.50**	**(1.14–1.98)**
High exposure (n = 977)	**1.32**	**(1.11–1.57)**	**1.66**	**(1.20–2.29)**	**1.88**	**(1.26–2.81)**
Arms above shoulder						
Low exposure (n = 6802)	1	(1–1)	1	(1–1)	1	(1–1)
Moderate exposure (n = 1659)	1.02	(0.90–1.17)	1.04	(0.82–1.33)	1.06	(0.78–1.43)
High exposure (n = 293)	1.20	(0.90–1.59)	1.39	(0.83–2.34)	1.51	(0.79–2.89)
Repeated arm movement						
Low exposure (n = 6177)	1	(1–1)	1	(1–1)	1	(1–1)
Moderate exposure (n = 1466)	1.05	(0.93–1.18)	1.09	(0.87–1.36)	1.11	(0.84–1.47)
High exposure (n = 1089)	1.04	(0.89–1.21)	1.07	(0.81–1.41)	1.09	(0.77–1.54)
Squatting or lying on knees						
Low exposure (n = 7137)	1	(1–1)	1	(1–1)	1	(1–1)
Moderate exposure (n = 1424)	1.06	(0.92–1.21)	1.11	(0.87–1.42)	1.14	(0.84–1.55)
High exposure (n = 204)	0.93	(0.68–1.29)	0.88	(0.49–1.59)	0.86	(0.41–1.79)
Pushing or pulling						
Low exposure (n = 6504)	1	(1–1)	1	(1–1)	1	(1–1)
Moderate exposure (n = 1822)	0.93	(0.81–1.06)	0.87	(0.68–1.12)	0.85	(0.62–1.15)
High exposure (n = 405)	0.96	(0.75–1.25)	0.94	(0.59–1.49)	0.92	(0.51–1.65)
Carrying or lifting						
Low exposure (n = 5860)	1	(1–1)	1	(1–1)	1	(1–1)
Moderate exposure (n = 2329)	**1.36**	**(1.20–1.55)**	**1.75**	**(1.39–2.20)**	**2.01**	**(1.51–2.68)**
High exposure (n = 571)	1.18	(0.94–1.48)	1.35	(0.89–2.05)	1.46	(0.87–2.45)

Table 2. Shows the OR and 95% CI at each of the independent variables. The dependent variable WAS includes 4 levels; poor (0) moderate (1) good (2) and excellent (3). The OR signifies the odds of having poor (0), moderate (1) or good (2) workability compared to excellent workability (3) adjusted for all other variables. The model also adjusted for gender, BMI, stress, mental health, smoking, leisure-time physical activity, depression, COPD, asthma, metabolic disease, cancer, diabetes and cardiovascular disease. Statistically significant differences (*p* < 0.05) from the base level are marked in bold.

## Data Availability

The data presented in this study are available on request from the corresponding author. The data are not publicly available due to privacy and ethical reasons.

## References

[1] World Health Organization (2016). Ageing and Life-Course. https://www.who.int/health-topics/ageing#tab=tab_1.

[2] Bohle P., Pitts C., Quinlan M. (2010). Time to call it quits? The safety and health of older workers. Int. J. Health Serv..

[3] Diehr P.H., Thielke S.M., Newman A.B., Hirsch C., Tracy R. (2013). Decline in Health for Older Adults: Five-Year Change in 13 Key Measures of Standardized Health. J. Gerontol. Ser. A.

[4] Goodpaster B.H., Park S.W., Harris T.B., Kritchevsky S.B., Nevitt M., Schwartz A.V., Simonsick E.M., Tylavsky F.A., Visser M., Newman A.B. (2006). The loss of skeletal muscle strength, mass, and quality in older adults: The health, aging and body composition study. J. Gerontol. A Biol. Sci. Med. Sci..

[5] Janssen I., Heymsfield S.B., Wang Z.M., Ross R. (2000). Skeletal muscle mass and distribution in 468 men and women aged 18–88 yr. J. Appl. Physiol. (1985).

[6] McMahan S., Sturz D. (2006). Implications for an Aging Workforce. J. Educ. Bus..

[7] Von Bonsdorff M.B., Seitsamo J., Ilmarinen J., Nygård C.L., von Bonsdorff M.E., Rantanen T. (2011). Work ability in midlife as a predictor of mortality and disability in later life: A 28-year prospective follow-up study. Can. Med. Assoc. J..

[8] Zwerling C., Sprince N.L., Wallace R.B., Davis C.S., Whitten P.S., Heeringa S.G. (1996). Risk factors for occupational injuries among older workers: An analysis of the health and retirement study. Am. J. Public Health.

[9] Søgaard K., Sjøgaard G. (2017). Physical Activity as Cause and Cure of Muscular Pain: Evidence of Underlying Mechanisms. Exerc. Sport Sci. Rev..

[10] Kinnunen U., Nätti J. (2018). Work ability score and future work ability as predictors of register-based disability pension and long-term sickness absence: A three-year follow-up study. Scand. J. Public Health.

[11] Van den Berg T.I.J., Elders L.A.M., de Zwart B.C.H., Burdorf A. (2009). The effects of work-related and individual factors on the Work Ability Index: A systematic review. Occup. Environ. Med..

[12] Ilmarinen J. (2006). Towards a longer and better working life: A challenge of work force ageing. Med. Lav..

[13] Ilmarinen J. (2009). Work ability—A comprehensive concept for occupational health research and prevention. Scand. J. Work Environ. Health.

[14] El Fassi M., Bocquet V., Majery N., Lair M.L., Couffignal S., Mairiaux P. (2013). Work ability assessment in a worker population: Comparison and determinants of Work Ability Index and Work Ability score. BMC Public Health.

[15] Leijten F.R.M., van den Heuvel S.G., Ybema j., van der Beek A.J., Robroek S.J.W., Burdorf A. (2014). The influence of chronic health problems on work ability and productivity at work: A longitudinal study among older employees. Scand. J. Work Environ. Health.

[16] Andersen L.L., Izquierdo M., Sundstrup E. (2017). Overweight and obesity are progressively associated with lower work ability in the general working population: Cross-sectional study among 10,000 adults. Int. Arch. Occup. Environ. Health.

[17] Calatayud J., Jakobsen M.D., Sundstrup E., Casaña J., Andersen L.L. (2015). Dose-response association between leisure time physical activity and work ability: Cross-sectional study among 3000 workers. Scand. J. Public Health.

[18] Andersen L.L., Mortensen O.S., Hansen J.V., Burr H. (2011). A prospective cohort study on severe pain as a risk factor for long-term sickness absence in blue- and white-collar workers. Occup. Environ. Med..

[19] Da Costa B.R., Vieira E.R. (2010). Risk factors for work-related musculoskeletal disorders: A systematic review of recent longitudinal studies. Am. J. Ind. Med..

[20] Andersen L.L., Clausen T., Burr H., Holtermann A. (2012). Threshold of musculoskeletal pain intensity for increased risk of long-term sickness absence among female healthcare workers in eldercare. PLoS ONE.

[21] Salisbury C., Johnson L., Purdy S., Valderas J.M., Montgomery A.A. (2011). Epidemiology and impact of multimorbidity in primary care: A retrospective cohort study. Br. J. Gen. Pract..

[22] Converso D., Sottimano I., Guidetti G., Loera B., Cortini M., Viotti S. (2018). Aging and Work Ability: The Moderating Role of Job and Personal Resources. Front. Psychol..

[23] Crawford J.D.A. (2020). Work-Related Musculoskeletal Disorders: Why Are They Still So Prevalent? Evidence from a Literature Review; Europoean Risk Obervatory Report.

[24] McDonald M., DiBonaventura M., Ullman S. (2011). Musculoskeletal Pain in the Workforce: The Effects of Back, Arthritis, and Fibromyalgia Pain on Quality of Life and Work Productivity. J. Occup. Environ. Med..

[25] Haukka E., Kaila-Kangas L., Ojajärvi A., Saastamoinen P., Holtermann A., Jørgensen M.B., Karppinen J., Heliövaara M., Leino-Arjas P. (2015). Multisite musculoskeletal pain predicts medically certified disability retirement among Finns. Eur. J. Pain.

[26] Oliv S., Noor A., Gustafsson E., Hagberg M. (2017). A Lower Level of Physically Demanding Work Is Associated with Excellent Work Ability in Men and Women with Neck Pain in Different Age Groups. Saf. Health Work..

[27] Bayattork M., Jakobsen M.D., Sundstrup E., Seidi F., Bay H., Andersen L.L. (2019). Musculoskeletal pain in multiple body sites and work ability in the general working population: Cross-sectional study among 10,000 wage earners. Scand. J. Pain.

[28] Truxillo D.M., Cadiz D.M., Rineer J.R., Zaniboni S., Fraccaroli F. (2012). A lifespan perspective on job design: Fitting the job and the worker to promote job satisfaction, engagement, and performance. Organ. Psychol. Rev..

[29] McGonagle A.K., Fisher G.G., Barnes-Farrell J.L., Grosch J.W. (2015). Individual and work factors related to perceived work ability and labor force outcomes. J. Appl. Psychol..

[30] Skovlund S.V., Bláfoss R., Sundstrup E., Andersen L.L. (2020). Association between physical work demands and work ability in workers with musculoskeletal pain: Cross-sectional study. BMC Musculoskelet. Disord..

[31] Andersen L.L., Burdorf A., Fallentin N., Persson R., Jakobsen M.D., Mortensen O.S., Clausen T., Holtermann A. (2014). Patient transfers and assistive devices: Prospective cohort study on the risk for occupational back injury among healthcare workers. Scand. J. Work Environ. Health.

[32] Neupane S., Miranda H., Virtanen P., Siukola A., Nygård C.L. (2013). Do physical or psychosocial factors at work predict multi-site musculoskeletal pain? A 4-year follow-up study in an industrial population. Int. Arch. Occup. Environ. Health.

[33] Punnett L., Gold J., Katz J.N., Gore R., Wegman D.H. (2004). Ergonomic Stressors and Upper Extremity Musculoskeletal Disorders in Automobile Manufacturing: A One Year Follow up Study. Occup. Environ. Med..

[34] Jensen L.K., Rytter S., Marott J.L., Bonde J.P. (2012). Relationship between years in the trade and the development of radiographic knee osteoarthritis and MRI-detected meniscal tears and bursitis in floor layers. A cross-sectional study of a historical cohort. BMJ Open.

[35] Kenny G.P., Yardley J.E., Martineau L., Jay O. (2008). Physical work capacity in older adults: Implications for the aging worker. Am. J. Ind. Med..

[36] Ilmarinen J., Tuomi K., Klockars M. (1997). Changes in the work ability of active employees over an 11-year period. Scand. J. Work Environ. Health.

[37] Oakman J., Neupane S., Proper K.I., Kinsman N., Nygård C.L. (2018). Workplace interventions to improve work ability: A systematic review and meta-analysis of their effectiveness. Scand. J. Work Environ. Health.

[38] Harris P.A., Taylor R., Thielke R., Payne J., Gonzalez N., Conde J.G. (2009). Research electronic data capture (REDCap)—A metadata-driven methodology and workflow process for providing translational research informatics support. J. Biomed. Inform..

[39] Ilmarinen J., Järvisalo J., Koskinen S. (2008). Dimensions of Work Ability—Results of the Health 2000 Survey.

[40] Kuorinka I., Jonsson B., Kilbom A., Vinterberg H., Biering-Sørensen F., Andersson G., Jørgensen K. (1987). Standardised Nordic questionnaires for the analysis of musculoskeletal symptoms. Appl. Ergon..

[41] Norheim K.L., Samani A., Bønløkke J.H., Omland Ø., Madeleine P. (2020). Physical performances show conflicting associations in aged manual workers. Sci. Rep..

[42] Chen H., Cohen P., Chen S. (2010). How Big is a Big Odds Ratio? Interpreting the Magnitudes of Odds Ratios in Epidemiological Studies. Commun. Stat. Simul. Comput..

[43] Statistics_Denmark (2021). RAS 201: Population (End November) by Region, Socioeconomic Status, Ancestry, Age And Sex. www.statistikbanken.dk/RAS201.

[44] Pohjonen T. (2001). Perceived work ability of home care workers in relation to individual and work-related factors in different age groups. Occup. Med..

[45] Tuomi K., Eskelinen L., Toikkanen J., Jarvinen E., Ilmarinen J., Klockars M. (1991). Work load and individual factors affecting work ability among aging municipal employees. Scand. J. Work Environ. Health.

[46] Monteiro M.S., Ilmarinen J., Filho H.R.C. (2006). Work ability of workers in different age groups in a public health institution in Brazil. Int. J. Occup. Saf. Ergon..

[47] Alavinia S.M., van den Berg T.I.J., van Duivenbooden C., Elders L.A.M., Burdorf A. (2009). Impact of work-related factors, lifestyle, and work ability on sickness absence among Dutch construction workers. Scand. J. Work Environ. Health.

[48] Sell L. (2009). Predicting long-term sickness absence and early retirement pension from self-reported work ability. Int. Arch. Occup. Environ. Health.

[49] Tuomi K., Ilmarinen J., Klockars M., Nygård C.H., Seitsamo J., Huuhtanen P., Martikainen R., Aalto L. (1997). Finnish research project on aging workers in 1981–1992. Scand. J. Work Environ. Health.

[50] Goedhard R.G., Goedhard W.J.A. (2005). Work ability and perceived work stress. Int. Congr. Ser..

[51] Van den Berg T.I.J., Alavinia S.M., Bredt F.J., Lindeboom D., Elders L.A.M., Burdorf A. (2008). The influence of psychosocial factors at work and life style on health and work ability among professional workers. Int. Arch. Occup. Environ. Health.

[52] Pensola T., Haukka E., Kaila-Kangas L., Neupane S., Leino-Arjas P. (2015). Good work ability despite multisite musculoskeletal pain? A study among occupationally active Finns. Scand. J. Public Health.

[53] Phongamwong C., Deema H. (2015). The impact of multi-site musculoskeletal pain on work ability among health care providers. J. Occup. Med. Toxicol..

[54] Miranda H., Kaila-Kangas L., Heliövaara M., Leino-Arjas P., Haukka E., Liira J., Viikari-Juntura E. (2010). Musculoskeletal pain at multiple sites and its effects on work ability in a general working population. Occup. Environ. Med..

[55] Kamaleri Y., Natvig B., Ihlebaek C.M., Bruusgaard D. (2008). Localized or widespread musculoskeletal pain: Does it matter?. Pain.

[56] Natvig B., Eriksen W., Bruusgaard D. (2002). Low back pain as a predictor of long-term work disability. Scand. J. Public Health.

[57] Urwin M., Symmons D., Allison T., Brammah T., Busby H., Roxby M., Simmons A., Williams G. (1998). Estimating the burden of musculoskeletal disorders in the community: The comparative prevalence of symptoms at different anatomical sites, and the relation to social deprivation. Ann. Rheum. Dis..

[58] Sim J., Lacey R.J., Lewis M. (2006). The impact of workplace risk factors on the occurrence of neck and upper limb pain: A general population study. BMC Public Health.

[59] Miranda H., Punnett L., Viikari-Juntura E., Heliövaara M., Knekt P. (2008). Physical work and chronic shoulder disorder. Results of a prospective population-based study. Ann. Rheum. Dis..

[60] Miranda H., Viikari-Juntura E., Heistaro S., Heliövaara M., Riihimäki H. (2005). A population study on differences in the determinants of a specific shoulder disorder versus nonspecific shoulder pain without clinical findings. Am. J. Epidemiol..

[61] Macfarlane G.J., Pallewatte N., Paudyal P., Blyth F.M., Coggon D., Crombez G., Linton S., Leino-Arjas P., Silman A.J., Smeets R.J. (2009). Evaluation of work-related psychosocial factors and regional musculoskeletal pain: Results from a EULAR Task Force. Ann. Rheum. Dis..

[62] Viikari-Juntura E., Martikainen R., Luukkonen R., Mutanen P., Takala E.P., Riihimäki H. (2001). Longitudinal study on work related and individual risk factors affecting radiating neck pain. Occup. Environ. Med..

[63] Linaker C.H., Walker-Bone K. (2015). Shoulder disorders and occupation. Best Pract. Res. Clin. Rheumatol..

[64] Sterud T. (2014). Work-related mechanical risk factors for long-term sick leave: A prospective study of the general working population in Norway. Eur. J. Public Health.

[65] Waters T.R., Dick R.B. (2015). Evidence of health risks associated with prolonged standing at work and intervention effectiveness. Rehabil. Nurs. Off. J. Assoc. Rehabil. Nurses.

[66] Lund T., Labriola M., Christensen K.B., Bültmann U., Villadsen E. (2006). Physical work environment risk factors for long term sickness absence: Prospective findings among a cohort of 5357 employees in Denmark. BMJ.

[67] McCulloch J. (2002). Health risks associated with prolonged standing. Work.

[68] Katzmarzyk P.T., Church T.S., Craig C.L., Bouchard C. (2009). Sitting time and mortality from all causes, cardiovascular disease, and cancer. Med. Sci. Sports Exerc..

[69] MacEwen B.T., MacDonald D.J., Burr J.F. (2015). A systematic review of standing and treadmill desks in the workplace. Prev. Med..

[70] Labriola M., Lund T., Burr H. (2006). Prospective study of physical and psychosocial risk factors for sickness absence. Occup. Med..

[71] Andersen L.L., Fallentin N., Thorsen S.V., Holtermann A. (2016). Physical workload and risk of long-term sickness absence in the general working population and among blue-collar workers: Prospective cohort study with register follow-up. Occup. Environ. Med..

[72] Frontera W.R., Hughes V.A., Lutz K.J., Evans W.J. (1991). A cross-sectional study of muscle strength and mass in 45- to 78-yr-old men and women. J. Appl. Physiol..

[73] Hawkins S., Wiswell R. (2003). Rate and mechanism of maximal oxygen consumption decline with aging: Implications for exercise training. Sports Med..

[74] Tuomi K., Ilmarinen J., Martikainen R., Aalto L., Klockars M. (1997). Aging, work, life-style and work ability among Finnish municipal workers in 1981–1992. Scand. J. Work. Environ. Health.

[75] Labriola M., Feveile H., Christensen K.B., Strøyer J., Lund T. (2009). The impact of ergonomic work environment exposures on the risk of disability pension: Prospective results from DWECS/DREAM. Ergonomics.

[76] Hulshof C.T.J., Pega F., Neupane S., van der Molen H.F., Colosio C., Daams J.G., Descatha A., Kc P., Kuijer P.P.F.M., Mandic-Rajcevic S. (2021). The prevalence of occupational exposure to ergonomic risk factors: A systematic review and meta-analysis from the WHO/ILO Joint Estimates of the Work-related Burden of Disease and Injury. Environ. Int..

[77] Wang X., Perry T.A., Arden N., Chen L., Parsons C.M., Cooper C., Gates L., Hunter D.J. (2020). Occupational Risk in Knee Osteoarthritis: A Systematic Review and Meta-Analysis of Observational Studies. Arthritis Care Res..

[78] Andersen L.L., Vinstrup J., Sundstrup E., Skovlund S.V., Villadsen E., Thorsen S.V. (2021). Combined ergonomic exposures and development of musculoskeletal pain in the general working population: A prospective cohort study. Scand. J. Work. Environ. Health.

[79] Roelen C.A.M., Heymans M.W., Twisk J.W.R., van der Klink J.J.L., Groothoff J.W., van Rhenen W. (2014). Work Ability Index as Tool to Identify Workers at Risk of Premature Work Exit. J. Occup. Rehabil..

[80] Ahlstrom L., Grimby-Ekman A., Hagberg M., Dellve L. (2010). The work ability index and single-item question: Associations with sick leave, symptoms, and health—A prospective study of women on long-term sick leave. Scand. J. Work Environ. Health.

[81] Barrero L.H., Katz J.N., Dennerlein J.T. (2009). Validity of self-reported mechanical demands for occupational epidemiologic research of musculoskeletal disorders. Scand. J. Work Environ. Health.

